# Systemic Therapies Following Progression on First-line CDK4/6-inhibitor Treatment: Analysis of Real-world Data

**DOI:** 10.1093/oncolo/oyac075

**Published:** 2022-04-25

**Authors:** James M Martin, Elizabeth A Handorf, Alberto J Montero, Lori J Goldstein

**Affiliations:** University Hospitals/Seidman Cancer Center, Case Western Reserve University, Cleveland, OH, USA; Fox Chase Cancer Center, Temple University, Philadelphia, PA, USA; University Hospitals/Seidman Cancer Center, Case Western Reserve University, Cleveland, OH, USA; Fox Chase Cancer Center, Temple University, Philadelphia, PA, USA

**Keywords:** CDK4/6 inhibitor, palbociclib, abemaciclib, ribociclib, everolimus

## Abstract

**Background:**

Metastatic hormone receptor positive (HR+)/human epidermal growth factor receptor-2 negative (Her2−) breast cancer remains a significant cause of cancer-related mortality. First-line treatment with endocrine therapy (ET) with a cyclin-dependent kinases 4 and 6 inhibitor (CDK4/6i) has largely become the standard systemic therapy. Following progression, no prospective randomized data exist to help guide second-line treatment.

**Materials and Methods:**

This study used a nationwide electronic health record (EHR)-derived de-identified database, specifically analyzing 1210 patients with HR+/Her2− metastatic breast cancer (MBC) who were treated in the first-line setting with a CDK4/6i from the years 2015-2020. The aim of this study was to assess what therapies were given after first-line progression on CDK4/6i and to observe treatment patterns over time. Determination of second-line treatment efficacy, specifically assessing real-world progression-free survival (rwPFS) and overall survival (OS) was performed.

**Results:**

A total of 839 patients received a documented second-line therapy after progression on first-line CDK4/6i treatment. Chemotherapy was chosen for 29.7% of patients, and the use of chemotherapy decreased over time. Three hundred two (36.0%) of patients continued a CDK4/6i. Data were adjusted for age, race, Eastern Cooperative Oncology Group (ECOG) performance status, stage at breast cancer diagnosis, and insurance payer type. Continuation of the CDK4/6i was associated with improved rwPFS (HR 0.48, 95% CI 0.43-0.53, *P* < .0001) and OS (HR 0.30, 95% CI 0.26-0.35, *P* < .0001) compared to chemotherapy. A majority of these patients continued the same CDK4/6i in the second-line setting, as was given in the first-line setting.

**Conclusion:**

While prospective data are needed, analysis of real-world data suggests a survival benefit for continuation of a CDK4/6i beyond frontline progression for patients with HR+/Her2− MBC.

## Teaser

Metastatic HR+/Her2– breast cancer is a significant cause of cancer-related mortality. First-line treatment with endocrine therapy with a cyclin-dependent kinases 4 and 6 inhibitor has become standard systemic therapy. Following progression, no prospective randomized data exist to help guide second-line treatment. This article aims to bridge that gap.

**Implications for Practice**

CDK4/6 inhibitors, in combination with ET, remain the standard of care first-line treatment for a majority of patients with HR+/Her2− metastatic breast cancer, and currently there are only minimal prospective data to guide treatment decisions following clinical progression on a CDK4/6 inhibitor. Continuation of the CDK4/6 inhibitor following first-line progression may be a reasonable tactic for some patients. Changing the ET partner could be considered (for example, changing from an aromatase inhibitor to fulvestrant). CDK4/6 inhibitors and everolimus appear to have a survival benefit over cytotoxic chemotherapy in the second-line setting.

## Introduction

Hormone receptor positive (HR+) breast cancers represent approximately 70%-80% of all breast cancers seen among women.^1,2^ Metastatic HR+/human epidermal growth factor receptor 2-negative (Her2−) breast cancer has been historically treated with sequential courses of endocrine therapy (ET) prior to the eventual need for cytotoxic chemotherapy.^3,4^ Attempts to both extend and enhance the efficacy of ET have led to the discovery and development of pharmacological inhibitors of the cyclin-dependent kinases 4 and 6 (CDK 4/6). CDK4/6, as well as their target protein cyclin D1, are involved in cell cycle regulation and have been implicated in the pathogenesis of breast cancer and potential development of endocrine resistance.^5,6^ Currently, there are 3 available CDK4/6 inhibitors (CDK4/6i) approved by the US Food and Drug Administration (FDA): palbociclib (Ibrance, Pfizer), ribociclib (Kisqali, Novartis), and abemaciclib (Verzenio, Eli Lilly).

Palbociclib was the first CDK4/6i to demonstrate a progression-free survival (PFS) benefit when administered with fulvestrant, compared to fulvestrant monotherapy, in patients who had progressed on prior ET.^7^ Both ribociclib and abemaciclib also showed a statistically significant PFS benefit for similar patient populations.^8,9^ Ultimately, this has led to improvements in overall survival (OS) in the second-line setting for patients treated with CDK4/6i.^10-12^ These agents are also approved in the first-line treatment setting in combination with an aromatase inhibitor (AI). All 3 drugs have again showed a statistically significant PFS benefit compared to AI monotherapy in this setting.^13-15^ ET with a CDK4/6i has largely become the standard of care initial therapy for patients with advanced HR+/Her2− breast cancer.

After progression on CDK4/6i therapy, no standard of care exists for the next line of systemic therapy. Reasonable options include switching to another ET monotherapy, cytotoxic chemotherapy, ET with everolimus (a mammalian target of rapamycin [mTOR] inhibitor), talazoparib or olaparib (poly(ADP-ribose) polymerase [PARP] inhibitors) for patients with germline *BRCA* mutations, or alpelisib (the phosphoinositide 3-kinase [PI3K] inhibitor) for patients with somatic *PIK3CA* mutations.^16^ Whether or not the CKD4/6i should be continued after initial disease progression is currently unknown. In an attempt to help fill this knowledge gap, we sought to analyze real world data to determine what systemic therapies were being used following progression on a CDK4/6i and compare differences in PFS and OS between the different treatments.

## Materials and Methods

The nationwide Flatiron Health electronic health record-derived de-identified database was used for this analysis. The Flatiron Health database is a longitudinal database, comprising de-identified patient-level structured and unstructured data, curated via technology-enabled abstraction.^17,18^ During the study period, the de-identified data originated from approximately 280 US cancer clinics (~800 sites of care). The majority of patients in the database originate from community oncology settings; relative community/academic proportions may vary depending on study cohort. We evaluated patient data collected from 2015 to 2020 for women with HR+/Her2− metastatic breast cancer (MBC) who received a CDK4/6i in combination with ET as first-line therapy and then received a documented second-line systemic therapy. The primary objectives of this study were to describe what systemic therapies were given as second-line treatment following first-line treatment with CDK4/6i, and to estimate the real-world PFS (rwPFS) and OS of those second-line therapies. rwPFS was defined as time between initiation of second-line therapy until clinician-recorded progression (or death). Patients who did not progress or die were considered censored at their last clinic visit. OS was defined as time between initiation of second-line therapy and death or end of follow-up (last confirmed activity), with patients alive at the end of follow-up considered censored. We assessed the relationship between patient characteristics and choice of second-line therapy type using T-tests or Chi-squared tests. We characterized OS and rwPFS using Kaplan-Meier curves with log-rank tests.^19,20^ We also assessed the relationships between survival and second-line therapy in multivariable Cox proportional hazards regression models, adjusting for age, race, Eastern Cooperative Oncology Group (ECOG) performance status (within a 3 month window of initiation of second-line therapy), stage at time of breast cancer diagnosis, insurance payer type, year of metastatic diagnosis, and months on first-line treatment. The main survival analyses were performed in the subset of patients who received second-line therapy. We also conducted a sensitivity analysis, including all patients in the cohort to account for patients who did not go on to receive second-line therapies and to address any potential differences in time between end of first-line therapy and the start of second-line therapy. For this analysis, we re-defined the start time as the time of documented progression on first-line therapy or receipt of the last dose of first-line therapy (if progression was not documented). We then entered second-line treatment as a time-varying covariate into the Cox model, with the effect of second-line treatment being the primary result of interest.

## Results

### Patient Characteristics

A total of 1210 patients with MBC received a CDK4/6i with ET as their documented first-line systemic therapy from years 2015-2020 (Table 1). Among these patients, 352 (29.2%) were documented as having presented with de novo metastatic disease, while the others had recurrent breast cancer following a prior diagnosis of early-stage disease. The mean age was 64.4 years (range 28-84). A majority of patients were white (69.4%) with an ECOG performance status of 0-1 (81.2%). Palbociclib was the most commonly used CKD4/6i in the first-line setting (88.2%), followed by ribociclib (7.2%) and abemaciclib (4.6%). A majority of patients received an AI as the ET partner (68.8%), although fulvestrant was used in nearly one-third of patients.

**Table 1.** Patient characteristics

	Overall (*N* = 1210)
Stage at Initial Diagnosis	
0	1 (0.1%)
I	147 (12.1%)
II	352 (29.2%)
III	268 (22.1%)
IV	352 (29.1%)
Age	
Mean	64.4 years
Range	28-84 years
Race	
Asian	39 (3.2%)
Black or African American	99 (8.2%)
Hispanic or Latino	3 (0.2%)
Other Race	129 (10.7%)
White	840 (69.4%)
ECOG Performance Status	
0	360 (37.2%)
1	426 (44.0%)
2	146 (15.1%)
3	33 (3.4%)
4	4 (0.4%)
First-Line CDK4/6i Used	
Palbociclib	1067 (88.2%)
Ribociclib	87 (7.2%)
Abemaciclib	56 (4.6%)
Endocrine Partner Used	
Anastrozole	59 (4.9%)
Exemestane	28 (2.3%)
Fulvestrant	366 (30.2%)
Letrozole	745 (61.6%)
Tamoxifen	12 (1.0%)

Abbreviations: ECOG, Eastern Cooperative Oncology Group; CDK4/6i, cyclin-dependent kinases 4/6 inhibitor.

### Treatment Characteristics

Eight hundred thirty-nine patients received a documented second-line systemic therapy (Table 2). Cytotoxic chemotherapy was the most common second-line therapy, followed by endocrine monotherapy, administered to 249 (29.7%) and 104 (12.4%) of patients, respectively. Fulvestrant was the most commonly prescribed endocrine monotherapy (70 patients, 8.3%). Other targeted therapies used were everolimus (99 patients, 11.7%), alpelisib (16 patients, 1.9%) and a PARP inhibitor (4 patients, 0.5%). Additionally, 51 (6.1%) patients were documented to enroll in a clinical trial for their second-line therapy, but no further data regarding the systemic therapy used were available.

**Table 2.** Second-line therapy used.

	Overall (*N* = 839)
AI	23 (2.7%)
CDK4/6i	4 (0.5%)
CDK4/6i + AI	97 (11.6%)
CDK4/6i + F	160 (19.1%)
CDK4/6i + F + AI	35 (4.2%)
CDK4/6i + F + T	3 (0.4%)
CDK4/6i + T	3 (0.4%)
Chemotherapy	249 (29.7%)
F	70 (8.3%)
F + AI	14 (1.7%)
Everolimus	99 (11.7%)
PARP Inhibitor	4 (0.5%)
Alpelisib	16 (1.9%)
T	11 (1.3%)
Clinical Trial	51 (6.1%)

Abbrevistions: AI, aromatase inhibitor; CDK4/6i, cyclin-dependent kinases 4/6 inhibitor; F, fulvestrant; T, tamoxifen; PARP, poly(ADP ribose) polymerase.

Three hundred eight patients received a CDK4/6i in the second-line setting, although 6 of these patients received chemotherapy in addition to a CDK4/6i and were subsequently grouped in the “chemotherapy” cohort for efficacy analysis. Thus, 302 (36.0%) patients were continued on a CDK4/6i in the second-line treatment setting, either alone or in combination with ET. The proportion of patients who received a CDK4/6i in the second-line setting increased over time, with patients initiating second-line treatment in later years having a higher rate of CDK4/6i use (*P* = .035). Conversely, the proportion of patients who received cytotoxic chemotherapy as second-line treatment appears to have decreased over time (*P* < .001).

Of the patients who received a CKD4/6i in the second-line setting, 229 (74.4%) received the same CDK4/6i that had been previously prescribed (Table 3). However, patients who received abemaciclib or ribociclib in the first-line setting were more likely to receive a different CDK4/6i than those who started with palbociclib (54.2% and 39.1%, respectively, vs. 21.8%). For the 261 patients who received palbociclib in the first-line setting, 204 (78.2%) continued palbociclib in the second-line setting, while 37 (14.2%) and 20 (7.7%) were switched to abemaciclib and ribociclib, respectively. For the 24 patients who received abemaciclib for first-line therapy, 11 (45.8%) continued abemaciclib, and 13 (54.2%) switched to palbociclib. Twenty-three patients received ribociclib first-line, and 14 (60.9%) of those were maintained on ribociclib in the second-line setting, while 1 (4.3%) and 8 (34.8%) were transitioned to abemaciclib and palbociclib, respectively. Among the 160 patients who received CDK4/6i with fulvestrant in the second-line setting, 81.2% of those received a CDK4/6i with an AI as first-line treatment.

**Table 3.** First-line versus second-line CDK4/6 used

	Abemaciclib (First-line, *N* = 24)	Palbociclib (First-line, *N* = 261)	Ribociclib (First-line, *N* = 23)	Total (*N* = 308)
Abemaciclib (Second-line)	11 (45.8%)	37 (14.2%)	1 (4.3%)	49 (15.9%)
Palbociclib (Second-line)	13 (54.2%)	204 (78.2%)	8 (34.8%)	225 (73.1%)
Ribociclib (Second-line)	0 (0.0%)	20 (7.7%)	14 (60.9%)	34 (11.0%)

### Efficacy Data

**Figure 1.**
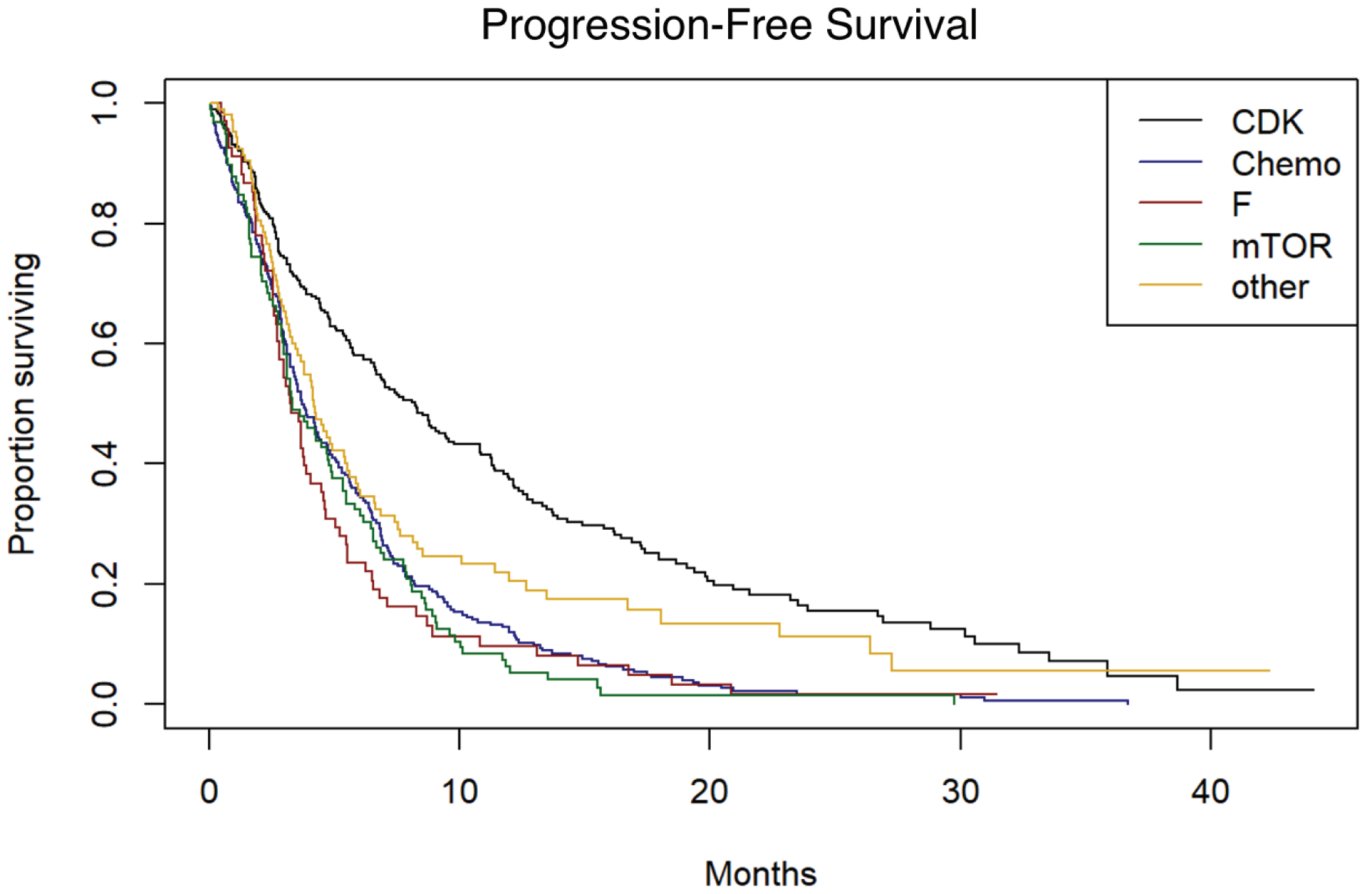
Real-world progression-free survival. CDK, cyclin-dependent kinases 4/6 inhibitors; Chemo, cytotoxic chemotherapy; F, fulvestrant; mTOR, mammalian target of rapamycin inhibitor (everolimus). “Other” includes endocrine therapies, various targeted therapies, trial drugs, etc. that do not fit into any of the other 4 categories.

**Figure 2.**
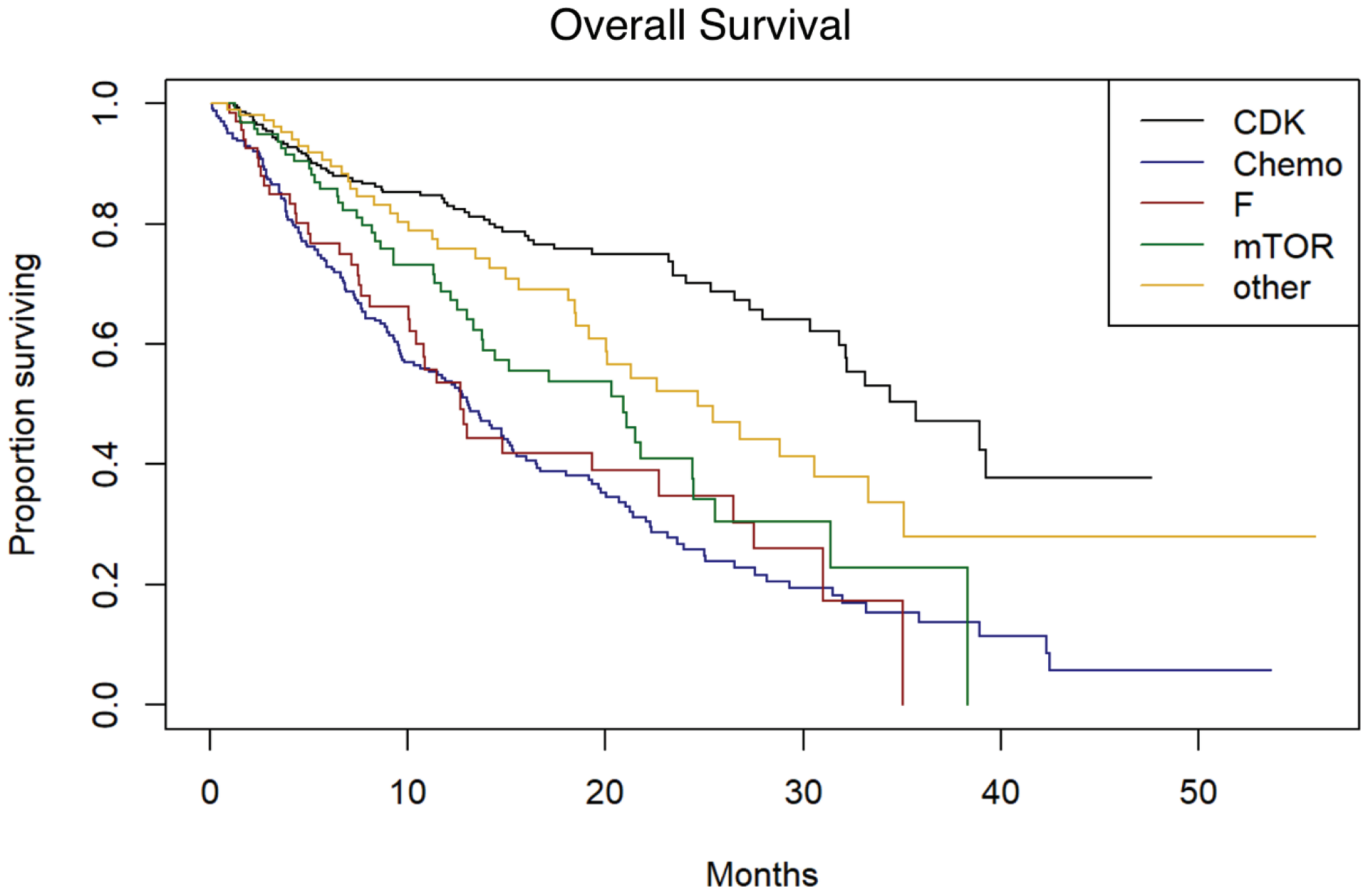
Overall survival. CDK, cyclin-dependent kinases 4/6 inhibitors; Chemo, cytotoxic chemotherapy; F, fulvestrant; mTOR, mammalian target of rapamycin inhibitor (everolimus). “Other” includes endocrine therapies, various targeted therapies, trial drugs, etc. that do not fit into any of the other 4 categories.

Real-world PFS (rwPFS) and OS were determined for the second-line treatments used in this cohort. Data were adjusted for age, race, ECOG performance status, stage at breast cancer diagnosis, insurance payer type, year of metastatic diagnosis and number of months on first-line treatment. The median unadjusted rwPFS and OS for those who received a CDK4/6i in the second-line setting were 8.25 months and 35.7 months, respectively (Figs. 1 and 2). The median rwPFS for those patients who received chemotherapy, fulvestrant monotherapy, or everolimus were 3.71, 3.25, and 3.32 months, respectively. On adjusted analysis, continuation of CDK4/6i was associated with a significantly improved rwPFS compared to chemotherapy (HR 0.48, 95% CI 0.43-0.53, *P* < .0001). Treatment with fulvestrant monotherapy or everolimus was not observed to have statistically significant benefits in rwPFS compared to chemotherapy. OS also favored continuation of the CDK4/6i (HR 0.30, 95% CI 0.26-0.35, *P* < .0001). Treatment with everolimus was also associated with improved OS compared to chemotherapy (HR 0.61, 95% CI 0.51-0.74, *P* = .0067), but fulvestrant was not. In a sensitivity analysis, accounting for patients who did not start second-line therapy, and re-defining the start of follow-up (as the time of end of first-line therapy or progression on first-line therapy) did not change the findings with respect to the effects of second-line therapy type.

## Discussion

The development and utilization of CDK4/6i have revolutionized the treatment paradigms for HR+/Her2− advanced or MBC. First-line use of these agents in combination with ET is endorsed by current National Comprehensive Cancer Network (NCCN) treatment guidelines.^21^ Our retrospective study suggests that continuation of CDK4/6i beyond first progression with different ET may be a therapeutically effective strategy as evidenced by a longer median rwPFS and OS, compared to chemotherapy or single-agent ET. Currently, there are no prospective data to suggest that continuation of CDK4/6i beyond initial progression is effective. However, a recent study reported data from a single institution of an estimated median PFS of 10.3 months with palbociclib-fulvestrant as a second-line therapy among patients who had progressed on palbociclib-AI.^22^ Additionally, in a multicenter cohort study, a subset of patients appeared to derive benefit from abemaciclib after clinical progression on a palbociclib-containing regimen.^23^ Prospective trials are needed to further address this therapeutic strategy, including whether the same CDK4/6i used in the first-line setting should be again used in the second-line setting.

Our study focused on second-line therapies after prior disease progression on a CDK4/6i in the first-line setting. We report a median OS of 35.7 months for patients who were treated with a CDK4/6i in the second-line setting. This compares favorably to OS values reported in the MONALEESA-3, MONARCH 2 and PALOMA-3 phase III trials, which compared a CDK4/6i in combination with fulvestrant to monotherapy with fulvestrant in previously treated patients, although prior receipt of a CDK4/6i was prohibited. Interestingly, nearly 5% of patients in this analysis received a CDK4/6i with dual ET (fulvestrant with either an AI or tamoxifen), which is not supported by prospective data. The clinical details regarding these therapeutic choices are unknown.

Our data also suggest that everolimus-based therapies may also portend improved OS compared to cytotoxic chemotherapy—a therapeutic strategy currently supported by the NCCN guidelines for second-line or later treatment of HR+/Her2− MBC. Real-world PFS was not improved with everolimus, possibly owing to more aggressive disease inherently belonging to the chemotherapy group, which may not allow for additional targeted agents and/or endocrine therapies to be attempted. The clinical characteristics of each treatment arm are unknown.

At this time, there are no prospective data to suggest that a particular patient with HR+/Her2− MBC would not benefit from the addition of a CDK4/6i to their initial treatment regimen, making these agents increasingly common within this population. Survival benefits have been noted across all clinicopathologic subgroups, including patients with progesterone receptor-negative disease, presence of visceral metastases, lobular histology, and many others.^24^ Additionally, abemaciclib appears to be biologically and pharmacodynamically distinct from palbociclib and ribociclib, with greater inhibition of CDK1 and CDK2.^25^ These differences may translate into unique clinical activities, but further data are needed.

The Flatiron Health database represents a useful means for assessing real-world practice patterns across the US. Moreover, the large number of patients with breast cancer in the dataset provides a unique opportunity to assess real world clinical outcomes, outside of the boundaries of a formalized clinical trial. “Real-world data” is defined by the FDA as healthcare information derived from atypical sources, such as health records, disease and product registries, and billing databases.^26^ Within the field of oncology, the publication of real-world data has markedly increased over time.^27^ With the rapidity of clinical progress and drug approvals recently in oncology, the use of real-world health data has the potential to help clinicians and researchers answer clinical questions for which there are no prospective clinical trial data.

There are some limitations to our study that should be considered. Since the data used were retrospective, we did not have access to several clinical characteristics of those patients who received a second-line treatment, such as patterns of their disease progression (sites of new metastases, severity of progression, presence of liver metastases or bone-only disease) or clinical status at the time progression. While the present study suggests some benefit to CDK4/6i continuation versus transitioning to cytotoxic chemotherapy, we should consider the possibility that those patients who received the latter may have had more aggressive disease (ie, visceral crisis), thus allowing for chemotherapy to be a more appropriate treatment modality. Conversely, it should be considered that the patients who continued on ET (with or without a targeted agent) may have had more indolent progressive disease. This level of data granularity is not available in our retrospective cohort. It also remains unclear from these data whether it would be more appropriate to continue the same CDK4/6i in the second-line setting, or switch to another CDK4/6i. In addition, we did not assess which particular chemotherapeutic agent was used for that subset of patients, as there appears to be differences in efficacy among the various agents following progression on ET.^28^

## Conclusion

In summary, our study suggests that a substantial portion of patients are continuing CDK4/6i therapy following first-line progression, despite that lack of prospective evidence supporting this approach. While there are ongoing clinical trials assessing this important question (NCT02632045, NCT04318223, NCT03854903), our real-world data analysis suggests some potential benefit to this approach regarding PFS and OS. Presuming the clinical scenario is appropriate for ongoing ET, continuation of a CDK4/6i may be an option for certain patients according to these data. Further efforts are needed to assess the best treatment approaches and drug sequencing for our patients living with advanced or metastatic HR+/Her2− endocrine-resistant breast cancer.

## Data Availability

The data underlying this article will be shared at reasonable request to the corresponding author.
